# Bioprinted hydrogels in bone regeneration: a bibliometric analysis

**DOI:** 10.3389/fphar.2025.1532629

**Published:** 2025-02-03

**Authors:** Huijie Zhang, Xiaoyu Li, Zhenyu Jia, Kun Jiao, Chen Liu, Zixiang Deng, Yushu Bai, Xianzhao Wei, Xiaoyi Zhou

**Affiliations:** ^1^ Department of Orthopedics, Shanghai Changhai Hospital, Shanghai, China; ^2^ Department of Orthopedics, General Hospital of Southern Theater Command, Guangzhou, China; ^3^ Department of Orthopedics, Shanghai Changzheng Hospital, Shanghai, China; ^4^ Department of Outpatient Service, Military District Shenyang No. 1 Retreat Center for Separated Cadres, Liaoning, China

**Keywords:** bibliometric, bone regeneration, tissue engineering, hydrogel, bioprinting

## Abstract

**Background:**

The application of bioprinted hydrogels in the field of bone regeneration is garnering increasing attention. The objective of this study is to provide a comprehensive overview of the current research status, hotspots and research directions in this field through bibliometric methods, and to predict the development trend of this field.

**Methods:**

A search was conducted on 27 December 2024, for papers published on the Web of Science from 2010 to 2025. We used the bibliometrix package in the software program R to analyze the retrieved data and VOSviewer and CiteSpace to visualize hotspots and research trends in bioprinted hydrogels for bone regeneration.

**Results:**

We identified and reviewed 684 articles published in this field between 2010 and 2025. A total of 811 institutions and 1,166 researchers from 41 countries/regions contributed to these publications. Among them, China led in terms of the number of articles published, single-country publications (SCP), and multi-country publications (MCP). Our bibliometric-based visualization analysis revealed that the mechanical properties and osteogenic differentiation capacity of biomaterials have been a focal research topic over the past decade, while emerging research has also concentrated on the *in vitro* fabrication of stem cells for bone regeneration and osteogenic differentiation, particularly the precise application of *in situ* stem cell-loaded bioprinted organoids.

**Conclusion:**

This study provides an in-depth analysis of the research trajectory in the application of bioprinted hydrogels for bone regeneration. The number of research papers in this field is increasing annually, and the main research hotspots include bone regeneration, 3D printing, scaffolds, and hydrogels. Future research directions may focus on gelatin, additive manufacturing, and growth factors. Additionally, international collaboration is essential to enhance the effectiveness of bioprinted hydrogels in bone regeneration applications.

## 1 Introduction

The skeleton is a critical organ that provides structural support and facilitates movement within the body (Song et al., 2024). The primary etiologies of bone defects are fractures resulting from accidents and sports activities, resection of osseous tumors, and revision of prosthetic devices (Song et al., 2024). With the development of biomedical engineering, scientists are gradually focusing on treating bone defects through bone tissue engineering. An increasing number of researchers have conducted multidimensional studies on the human skeleton to identify effective therapeutic strategies for bone defects (Rosset et al., 2014), such as autologous bone grafting, allogeneic bone grafting, and a the use of artificial bone scaffolds (Zhu et al., 2017). Currently, autologous bone grafting is considered the “gold standard” for treating bone defects (Keating et al., 2005). However, autologous bone grafting is limited by bone volume, risk of infection, potential for secondary injury, and chronic pain (García-Gareta et al., 2015). Allogeneic bone grafts are also associated with complications such as infection and immune rejection (McEwan et al., 2018). Therefore, a less invasive treatment strategy that can consistently support bone regeneration over time is needed. Bone tissue engineering encompasses the application of *in vitro* cultured cells or growth factors onto biocompatible scaffolds, which are then deployed to target bone defects. This approach facilitates the release of cells and growth factors, promoting osteoblast proliferation and bone tissue regeneration (Shan and Wu, 2024). The selection of biomaterials, such as polymer scaffolds, bioactive glass, and hydrogels, is crucial for the success rate in healing bone defects. Key properties include excellent biocompatibility, biodegradability, high mechanical strength, and the ability to support cell adhesion and growth (Albrektsson and Johansson, 2001). Regarding the materials used for bone generation, the current focus is on metal, bioceramic, and polymer scaffolds (Wang et al., 2020). Metallic materials such as stainless steel, cobalt-chromium alloys, and titanium alloys are widely used in orthopedics and dentistry due to their exceptional mechanical strength (Iatecola et al., 2021); however, their poor biodegradability prevents them from being used as carriers of cells or growth factors in the field of bone regeneration (Tzagiollari et al., 2022). Bioceramic scaffolds, such as calcium phosphate (CaP), calcium sulfate, and calcium silicate (Wang et al., 2020), have entered the orthopedic field owing to their good biocompatibility, corrosion resistance, and low cost. Nevertheless, their inadequate fatigue resistance prevents them from serving as long-term bone tissue support within the human body. Therefore, hydrogels stand out among many biomaterials owing to their cell-loaded and tunable mechanical properties (Yue et al., 2020), and they have emerged as a significant component in regenerative medicine (Liu and Hsu, 2018).

**FIGURE 1:**
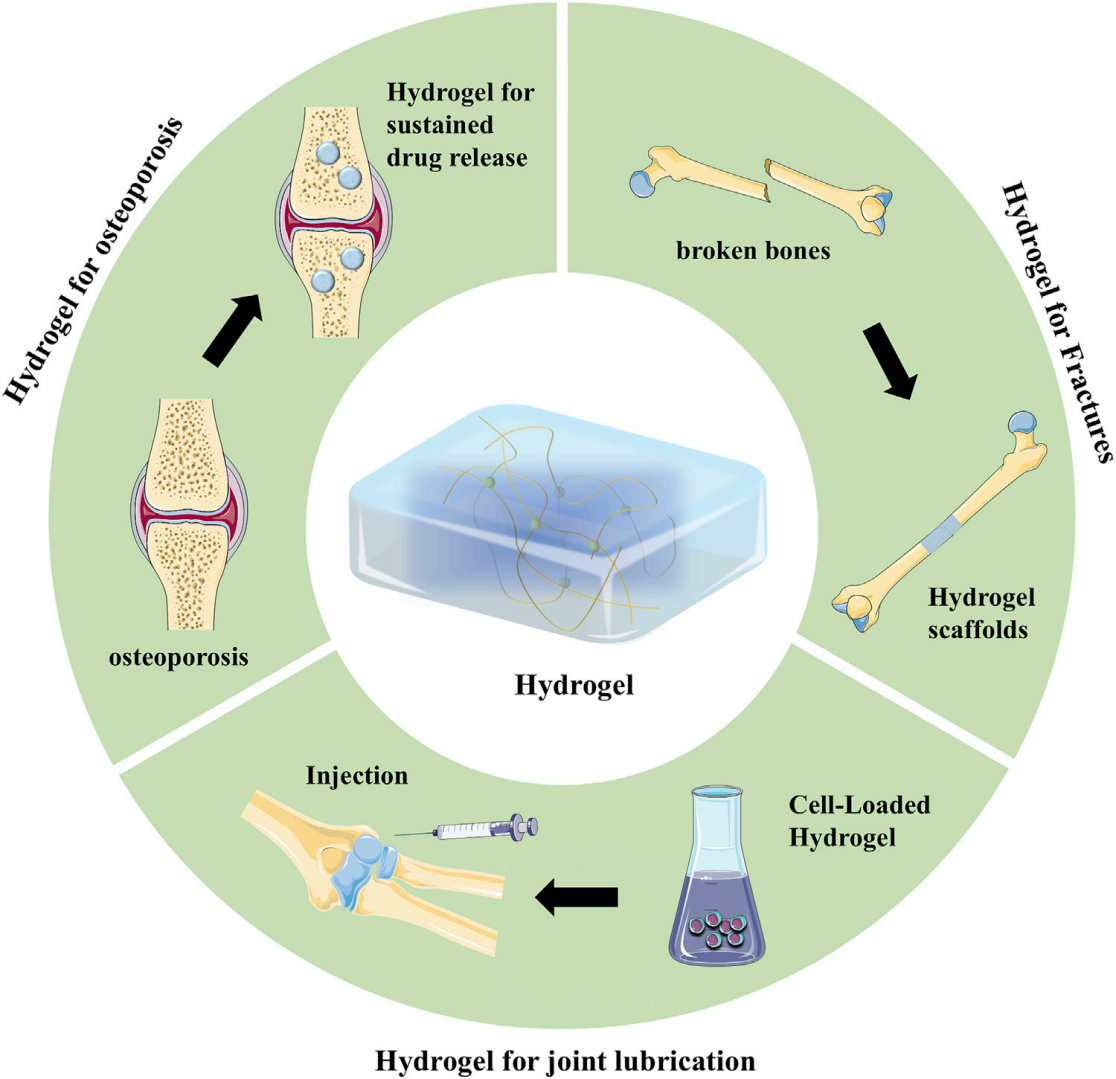
Hydrogels in bone regeneration.

Hydrogel is a polymer with a three-dimensional mesh structure, and its properties such as biocompatibility, nontoxicity, and degradability have led to a wide range of biomedical applications, such as wound dressings, contact lenses, and biosensors (Ortega et al., 2023; Cao et al., 2021). Polymer networks can be molded into three-dimensional structures with different porosities; therefore, they can provide constructive microenvironments suitable for controlled cell growth (Muir and Burdick, 2021; Zhang Y. et al., 2020; Oliva et al., 2021). Hydrogels have become excellent bioinks for bioprinting because of their high water content and three-dimensional mesh structure, which minimizes the shear stress on cells (Bertsch et al., 2023). Moreover, the high water content makes hydrogels highly permeable and porous, enabling the rapid diffusion of balanced oxygen and nutrients (Chimene et al., 2016). The tunable properties of hydrogels permit the adjustment of their mechanical strength to align with the specific demands of the target tissue (Li et al., 2018), enhancing applicability. Bioprinted hydrogels have become an important technology in the field of bone regeneration because their internal network structure can be modulated by changing the external geometry and volume of the scaffold to achieve dynamic drug delivery and fostering osteoblast development within the human body (Wan et al., 2020). As Figure 1 shows, hydrogels have a wide variety of applications in the bone regeneration field (Nie et al., 2019). With fractures, hydrogel scaffolds accelerate bone healing by releasing growth factors and have emerged as bone graft substitutes (Chen L. et al., 2023; Kolambkar et al., 2011). Hydrogels can utilize their degradation properties for the sustained release of drugs to enhance patients’ bone density and treat osteoporosis symptoms (Gong et al., 2022; Ding et al., 2023). Hydrogels serve as joint lubricants, effectively treating conditions such as osteoarthritis (Vinikoor et al., 2023; Duan et al., 2023). In minimally invasive surgery, hydrogels can be administered via injection to completely fill and address bone defect cavities (Ghandforoushan et al., 2023). Moreover, hydrogels are important for targeted drug delivery to inflammatory bone lesions (Xie et al., 2022; Kuo et al., 2023). With the rapid development of printing technology, bioprinting hydrogel combines cells with hydrogel ink to create tissue-like structures through 3D printing technology, providing a good environment for human tissues and cells to grow and develop (Zhu et al., 2022). Although hydrogels are somewhat uncontrollable in terms of degradation properties, drug release rate and mechanical properties, researchers in various fields are constantly trying new syntheses to achieve the stability of various properties of hydrogels. Currently, bioprinted hydrogels are predominantly utilized in the fields of skin tissue engineering (Zhou et al., 2020), cardiovascular tissue engineering (Sousa et al., 2021) and bone tissue engineering (Zhang J. et al., 2020). Among them, in order to achieve high mineralization of bone tissue and proliferation of cellular diversity. Researchers are also continuously developing hydrogel bioinks that can match the biological properties of bone tissue. Tavares et al., 2021 used a GelMA/MSN CaP Dex hydrogel as a bioink to fabricate three-dimensional bone tissue containing osteogenic tissue using extrusion-based 3D printing technology. Cidonio et al. (2020) accomplished the *in vitro* and *in vivo* growth of bone mineral tissue by utilizing Laponite^®^-alginate-methylcellulose casting of human bone marrow stromal cells as a biocarrier. Yuan et al. (2021) used photo-crosslinked methacrylated gelatin in combination with silica nanoparticles to achieve rapid diffusion of internal stem cells and improve the osteogenic efficiency of stem cells. With the rapid development of the additive manufacturing industry, the high resolution of bioprinting has led to it becoming the dominant manufacturing technology in the medical field (Mandrycky et al., 2016). This technique is extensively utilized in bone tissue engineering, regenerative medicine, and medical device applications (Wan et al., 2020). Three-dimensional (3D) bioprinting for treating bone defects has been the focus in the field (Zhang et al., 2019a). Furthermore, with the development of bioprinting technology, four-dimensional (4D) bioprinting has also been developed, which adds a temporal dimension to 3D bioprinting. Specifically, in 4D bioprinting, changes in temperature or pH over time stimulate the print (Kang et al., 2022), altering its mechanical properties to accommodate the growth patterns of autogenous bone (Suo et al., 2018), thereby offering new possibilities for the fabrication of irregular bone constructs in clinics (Murphy and Atala, 2014).

**FIGURE 2:**
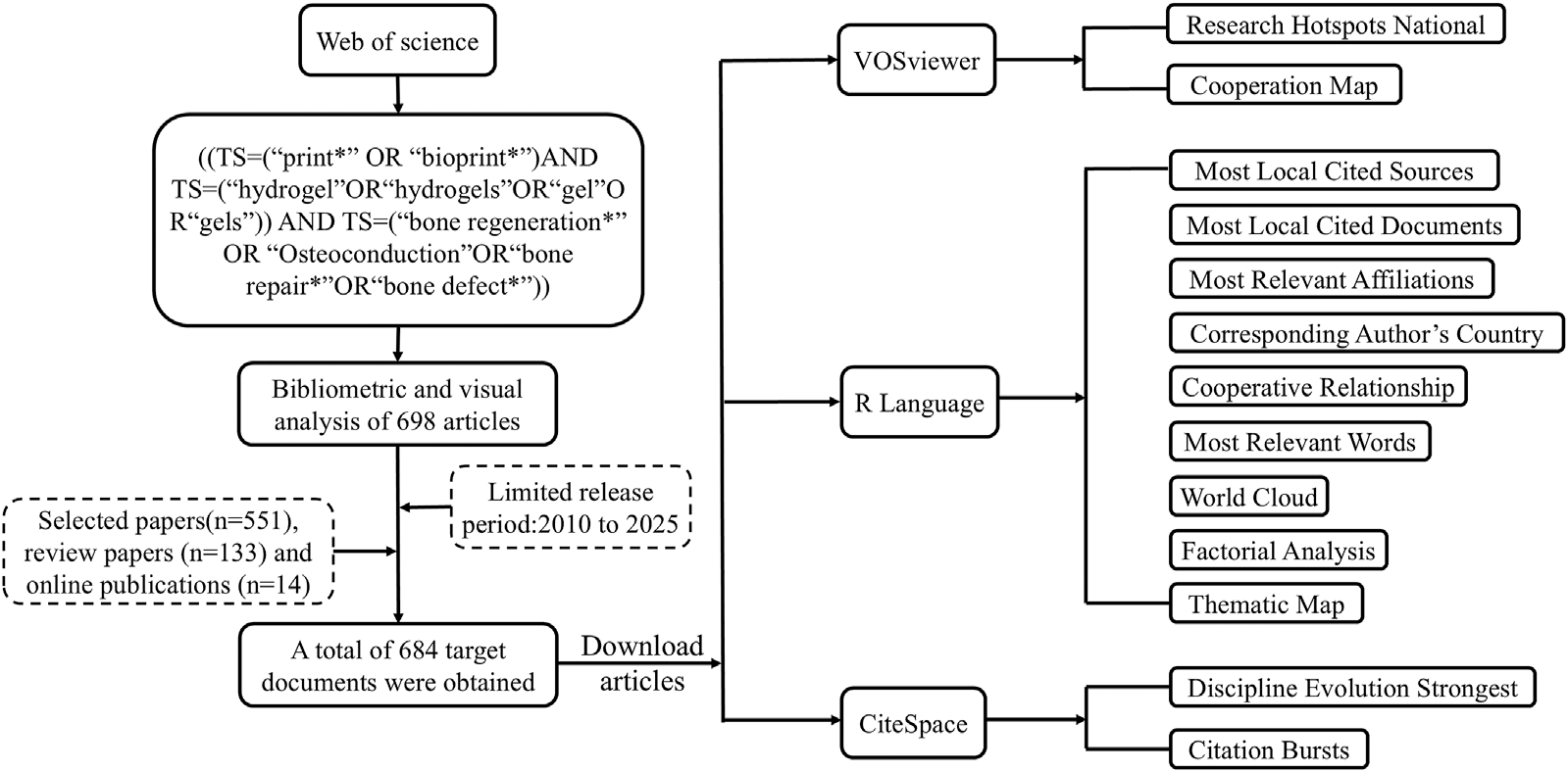
Graphical summary.

The discipline of bibliometrics is widely used to predict the direction of development and research patterns in a particular field (González-Alcaide et al., 2017). It employs different methods to assess research trends and helps researchers identify influential articles in the field, thereby contributing to continuously optimize research innovations (Bertsch et al., 2023; Zhang et al., 2024; Chen S. et al., 2023). By analyzing data such as the number of publications and the number of citations, it provides a reference basis for researchers who are about to enter the field to formulate their research plans. Through analyzing the cooperation network between countries and organizations, it assists researchers in the rational allocation of resources. Meanwhile, the construction of a knowledge map can help scholars quickly grasp the hot topics and research directions (Figure 2). In recent years, there has been a steady increase in the number of researchers focusing on bioprinted hydrogels for bone regeneration. However, There is a significant gap in the quantitative analysis of scientific results in this field, particularly concerning research trends, research quality, and the identification of interdisciplinary research gaps from a historical perspective. The objective of this paper is to evaluate the research articles on the application of bioprinted hydrogels in bone regeneration worldwide over the past 15 years by using the Web of Science core database and bibliometric tools to assess the current state of research, collaborative development paths, leading countries and institutions, cited literature, and future development trends according to the information on the distribution of publications by country, authors, journals, and impact. It is expected to help the subsequent researchers to understand the current research status in this field, formulating the systematic research strategy, and fostering the rapid development of bioprinted hydrogels in bone tissue engineering. These findings may assist subsequent researchers in understanding the current research landscape in this field, formulating systematic research strategies, and fostering the rapid advancement of bioprinted hydrogels in bone tissue engineering.

## 2 Materials and methods

### 2.1 Search strategies

The Web of Science database covers the largest amount of literature in the medical field and is frequently utilized in bibliometric studies (Wang et al., 2022). We collected relevant literature from this database, focusing on the application of bioprinted hydrogels for bone regeneration. To ensure accuracy and consistency, we conducted a systematic search on 27 December 2024, for relevant literature included in the Web of Science database between January 2010 and March 2025. All retrievals were performed on the same day to prevent data bias due to updates in the Web of Science. Based on the previous statistics, we set the search strategy as follows: [(TS=(“print*” OR “bioprint*”) AND TS=(“hydrogel”OR“hydrogels”OR“gel”OR“gels”)) AND TS=(“bone regeneration*” OR “Osteoconduction”OR“bone repair*”OR“bone defect*”)]. Removing conference abstracts, book chapters, and reviews, we retrieved a total of 684 related papers.

### 2.2 Data collection and statistics

684 articles were imported into VOSviewer (1.6.19) and bibliometric analysis was performed using the bibliometrix package (4.1.3) in R (4.3.0). We used tools such as CiteSpace (6.3.1), VOSviewer (1.6.19), the bibliometrix package (4.1.3) in R (4.3.0), and PS (2020) to process, visualize, and analyze the data of the 684 documents collected from the Web of Science database. The counting method used for all analyses was full count. In addition, an online bibliometric analysis platform (https://bibliometric.com) was used to assist in the analysis of the intensity of cooperation between countries.

CiteSpace (6.3.1) is an analytical software program developed for bibliometrics that employs algorithms to automatically extract and analyze key information in a research area. The tool analyzes the scope and hotspots of research in a given field and identifies trends for improvement in related disciplines (Cheng et al., 2022). We used this tool to obtain citation biplot overlays to analyze keywords, references, clusters, and collaborative relationships between countries and institutions. We used the burst detector option to detect the first 30 keywords. VOSviewer (1.6.19) systematically collects and organizes basic information, including countries, institutions, authors, journal publications, and collaborative networks, and visualizes and analyzes the data (van Eck and Waltman, 2010). This software can extract the key information that researchers require from a wide range of literature to create co-citation and co-authorship networks (Chen et al., 2021). We used software to explore the temporal distribution and dynamic variability of keywords in the field and accurately reveal the evolutionary trends of hotspots in the research area. In order to analyze the research hotspots, the type of analysis was selected as co-occurrence, the unit of analysis was selected as keywords, and the analysis was carried out using the full count method.

The bibliometrix package (version 4.1.3) in R is a mapping tool designed for systematic analysis (Aria et al., 2021) that enables the mapping and analysis of country distribution maps, author publications, and keyword development. It also facilitates the identification of trending themes and milestones in the literature for publications in related research areas. The most relevant affiliations should be selected as options during the analysis. Thematic maps and factor analysis are employed to elucidate the components of the constitutional structure.

## 3 Results

### 3.1 Analysis of most locally cited documents and sources in the field

#### 3.1.1 Most locally cited sources

**FIGURE 3:**
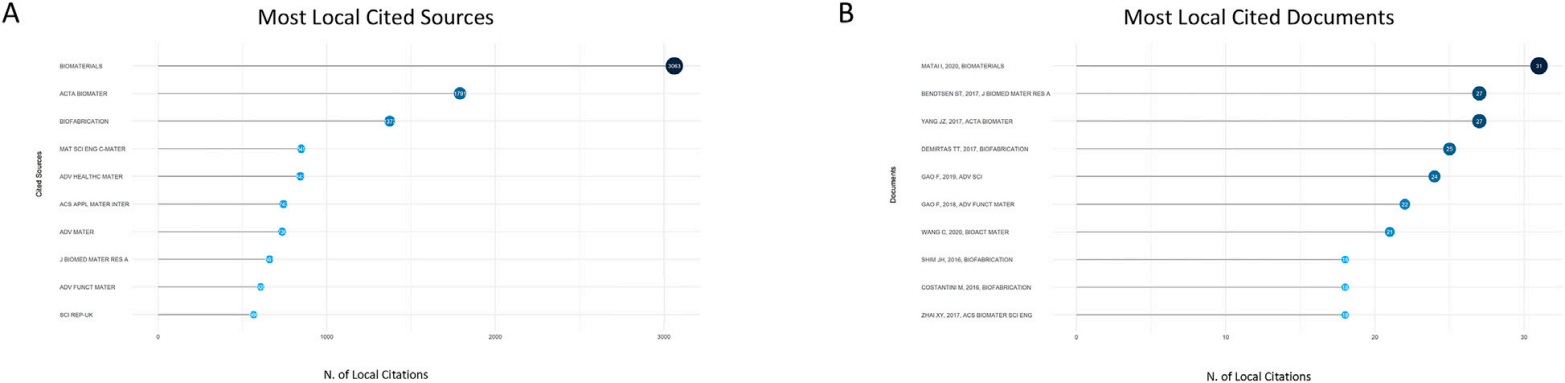
**(A)** The number of citations and the top ten highly cited journals in this field from 2006 to 2024. **(B)** The number of citations of highly cited documents in this field and the top ten articles from 2006 to 2024.

The 684 collected documents were analyzed in depth, which cited a total of 4,444 journals and were ranked according to the number of papers and the number of journals. The top ten journals in terms of the number of articles are shown in Figure 3A, with the most-cited journal being *Biomaterials* (3,488), which had far more citations than any other journal. This demonstrates that *Biomaterials* holds an authoritative position in the field of medical bioprinted hydrogels for bone regeneration and plays a significant role as trendsetters in the field.

#### 3.1.2 Most locally cited documents

The most locally cited literature can help researchers who are new to the field quickly select the best literature to read. The author rankings were based on the number of citations in the literature on bone regeneration with bioprinted hydrogel. Figure 3B illustrates the top ten cited papers in the field. TURNBULL G et al., ’s 2020 article in *Bioactive Materials*, “3D bioactive composite scaffolds for bone tissue engineering” was ranked first in citations and cited 38 times in this research area, with a total of cited 914 times.

### 3.2 Analysis of affiliations and countries

#### 3.2.1 Most relevant affiliation

A total of 811 research institutions, including universities, contributed to the literature published in the field. Table 1 lists the top 10 institutions in terms of the number of papers published, along with the name of the country where each institution is located. Supplementary Table S1 lists the top 10 authors with publications in the field. Sichuan University (SCU) in China ranked first with 92 research papers related to the application of bioprinted hydrogels in bone regeneration, followed by Shanghai Jiao Tong University (SJTU), also in China, with 53 papers. This demonstrates the outstanding research achievements of SCU and SJTU in the field of bioprinted hydrogels. The top 10 research institutions in this field are all affiliated with China, suggesting that Asia may be at a higher level of research in this field.

**TABLE 1:** Top 10 most relevant affiliations.

Rank	Institution	Contribution (%)	Country
1	Sichuan University	13.4	China
2	Shanghai Jiao Tong University	12.1	China
3	Zhejiang University	7.4	China
4	Chinese Academy of Sciences	7.1	China
5	Jilin University	5.8	China
6	South China University of Technology	5.1	China
7	Nanjing Medical University	4.8	China
8	Shandong University	4.5	China
9	China Medical University Taiwan	3.9	China
10	Jinan University	3.9	China

#### 3.2.2 Corresponding Author’s country

**FIGURE 4:**
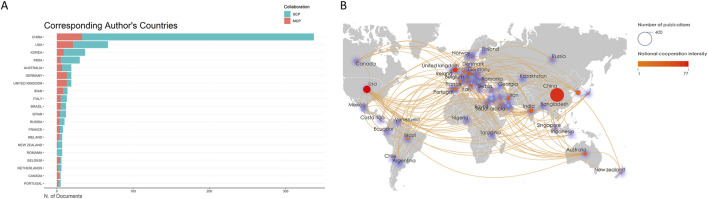
**(A)** Top 20 most productive countries. **(B)** Cooperation networks in countries around the world.

For single-country publications (SCPs), all authors of an article are all from the same country, whereas for multi-country publications (MCPs), the authors of an article are from more than one country, indicating international collaboration. As shown in Figure 4A, we analyzed the SCPs and MCPs of the top 20 countries. An analysis of the statistics on the nationalities of the corresponding authors of the relevant literature shows that China dominates this research field, with the United States ranking second. Among them, China’s SCP value has a clear gap with the United States, while China ranks first for MCPs. These data suggest that China is capable of conducting research in this field relatively independently and possesses a strong capacity for international cooperation. This result also corroborates those for major research institutions with the most relevant affiliations, with countries with a higher number of major research institutions also having more corresponding authors than other countries.

#### 3.2.3 Analysis of the cooperative relationship between countries

We filtered and visualized international cooperation based on the number of publications, constructing a network of cooperation based on the number of publications and relationships between countries. As shown in Figure 4B, the size of the circle radius indicates the country’s contribution to the number of papers in the field, the color of the circle indicates the intensity of the country’s cooperation, and the density of the lines around the circle indicates the number of collaborations between that country and other countries. The highest density of the lines indicates that the country is at the center of research in this field. Close cooperation has occurred between China and the United States, the United Kingdom, and Germany, while the United States has also engaged in productive cooperation with South Korea, the United Kingdom, and Iran, in addition to more cooperation with China.

### 3.3 Analysis of keywords and research hotspots

**FIGURE 5:**
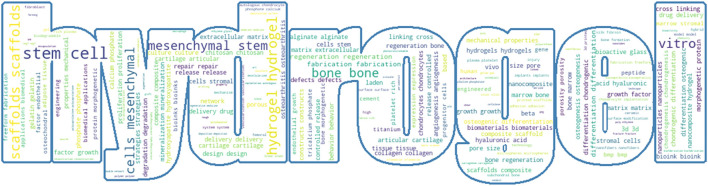
Wordcloud of the collection of literature keywords included in the study.

Keyword analysis can outline the research object, content, and hotspots within a research field by identifying the most frequent words in the field, which are central to academic papers. Supplementary Table S2 lists the top 10 keywords with the highest frequency in this field, and among the extensive literature, “bone regeneration”, “3D printing”, “scaffold” and “hydrogel” are the keywords that appear more frequently (Figure 5), indicating that bioprinted hydrogel scaffolds are the current research hotspot in the field of bone regeneration.

**FIGURE 6:**
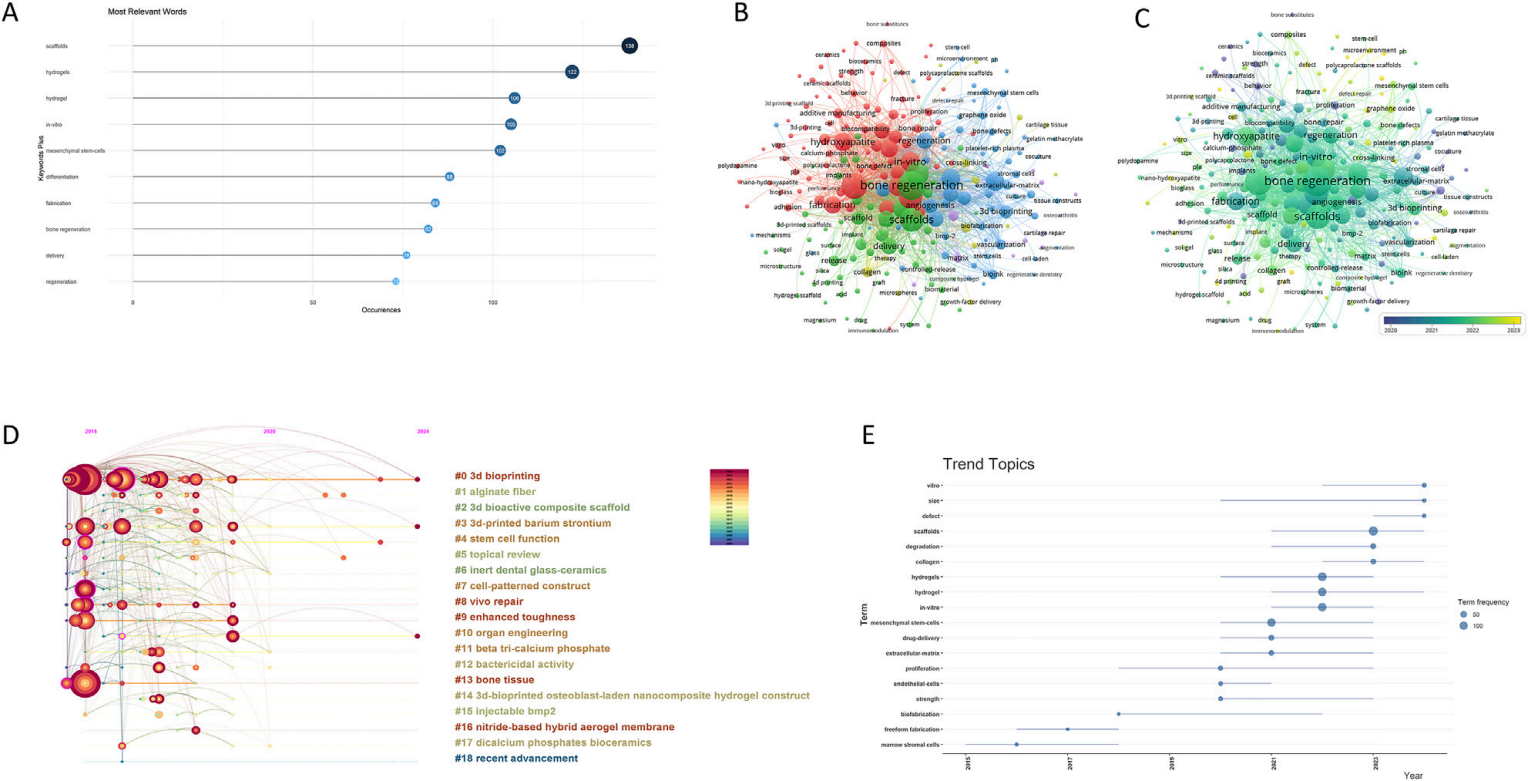
Analysis of keywords and Research Hotspots. **(A)** Top 10 most relevant words. **(B)** The keywords related to the application of bioprinted hydrogels in bone regeneration are divided into X clusters according to different colors. The size of the circle represents the frequency with which keywords appear. **(C)** The distribution of keywords is presented according to the average time of appearance. Purple keywords appear earlier than yellow keywords. **(D)** The timeline of clustering for keywords. **(E)** Map of keywords trend topics.

We analyzed the titles, subject headings, and abstracts of 684 papers to identify common phrases and determine their frequency of occurrence. As shown in Figure 6A, “scaffolds” (138) appears most frequently, which is consistent with the results of the word cloud search.

The keywords in the field were categorized into five clusters using VOSviewer, as shown in Figure 6B. Cluster 1 focuses on the development of 3D-printed hydrogel scaffolds in additive manufacturing. Cluster 2 mainly includes the application of cellular scaffolds in bone tissue engineering. Cluster 3 focuses on bio-inks such as hydrogels and the effects of biomaterials on cells in the microenvironment. Cluster 4 investigates the application of growth factors in the field of bone regeneration. Cluster 5 provides an overview of the current state-of-the-art research on 4D printing in the field of biomedical sciences.

We then further used VOSviewer to color-code all keywords based on the average publication year, revealing trends in the field over time and exploring upcoming innovation directions. By analyzing the keyword sequencing map (Figure 6C), we found that osteogenesis, microenvironment, hydrogel, and 4D printing are relatively novel and promising areas in the field of bone regeneration. Recently, researchers have combined bioprinted hydrogel technology with cell-loaded scaffolds to advance the field of bone regeneration by restoring microenvironmental homeostasis at bone defect sites.

Keyword clustering analysis reveals the main themes and development states of a particular research area (Zhong et al., 2020). CiteSpace was used to divide the keywords into 19 sets and generate a clustering timeline. As shown in Figure 6D, the keywords included “#0 3 d printing”“#1 alginate fiber”“#2 3d bioactive composite scaffold”“#3 3d-printed barium strontium”“#4 stem cell function”“#5 topical review”“#6 inert dental glass-ceramics”“#7 cell-patterned construct”“#8 vivo repair”“#9 enhanced toughness”“#10 organ engineering”“#11 beta tri-calcium phosphate”“#12 bactericidal activity”“#13 bone tissue”“#14 3 d-bioprinted osteoblast-laden nanocomposite hydrogel construct”“#15 injectable bmp2”“#16 nitride-based hybrid aerogel membrane”“#17 dicalcium phosphates bioceramics”and“#18 recent advancement”. That the result shows that “3D printing” is the most important research area for hydrogels in bone tissue engineering. To further confirm the accuracy of our results, we conducted a thematic analysis of keyword trends using R (Figure 6E). Keywords such as “microenvironment” “defect” “biomaterials” “cells” “osteogenesis” “scaffolds” have been emerging in the field recently, indicating their importance. In addition, “scaffolds” “mesenchymal stem-cells” “ vitro” “hyaluronic-acid hydrogels” and “biofabrication” are important parts of bone tissue engineering in long-term studies. The evolutionary trend of these themes is consistent with the results of our analysis above.

### 3.4 Thematic map

**FIGURE 7:**
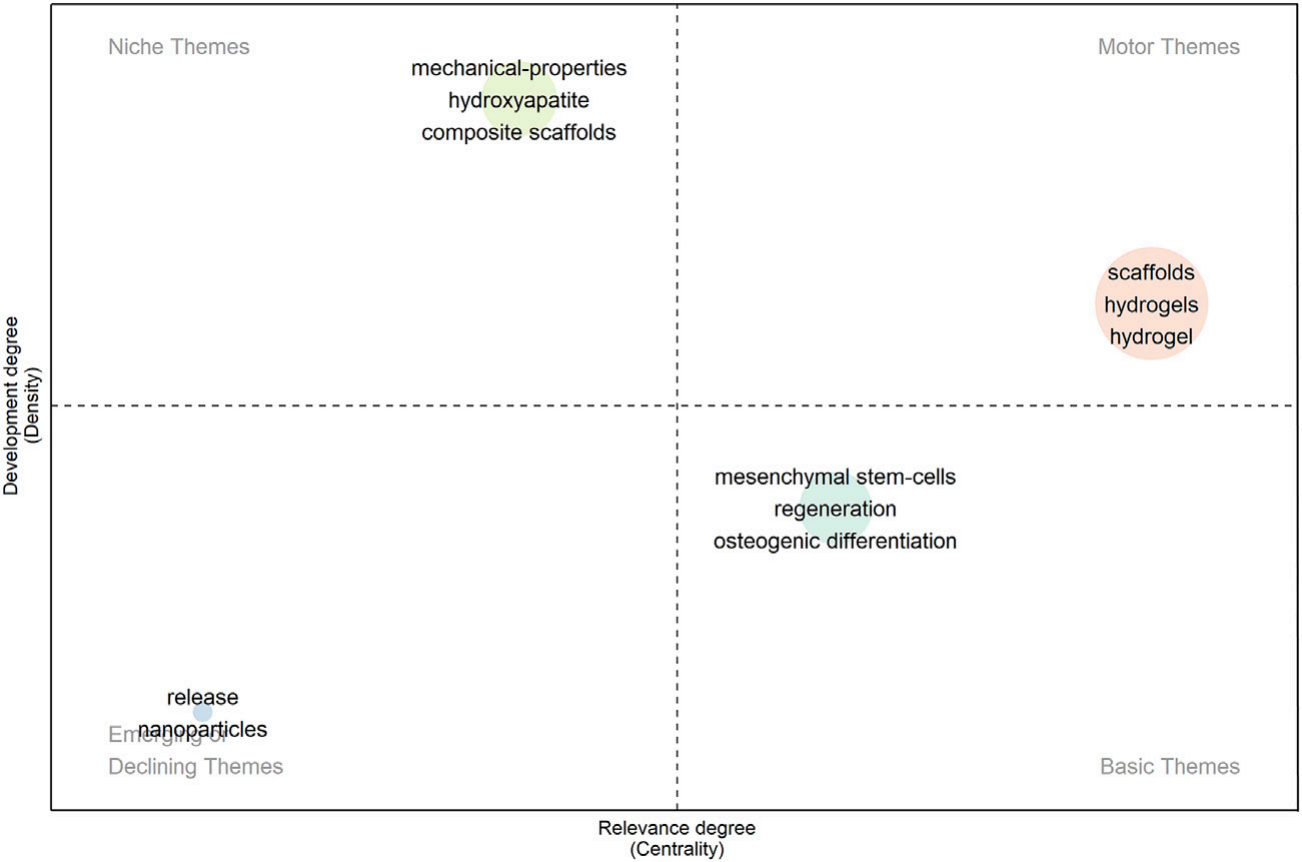
Strategic theme map.

Each quadrant on the thematic map has a specific meaning. The horizontal axis (*X*-axis) indicates centrality, and the vertical axis (*Y*-axis) indicates density. Regarding the quadrant distribution plotted in this study (Figure 7), the first quadrant (upper right) focuses on the optimization of hydrogel scaffolds, suggesting a promising future for this area. The second quadrant (upper left) shows the preparation of hydroxyapatite, the mechanical properties of biomaterials and the study of composite scaffolds, suggesting that this topic is well-developed but less important in the field of bone regeneration. The third quadrant (lower left) focuses on the imminent rapid surge or imminent slow disappearance of the controlled release of nanoparticles. Finally, the fourth quadrant (lower right) is dedicated to *in vitro* manufacturing of stem cells for bone regeneration and osteogenic differentiation. While these directions have not yet achieved mature research results, they hold an important position in the field of bone regeneration and may become a hot research area in the future.

### 3.5 Trends in discipline evolution

**FIGURE 8:**
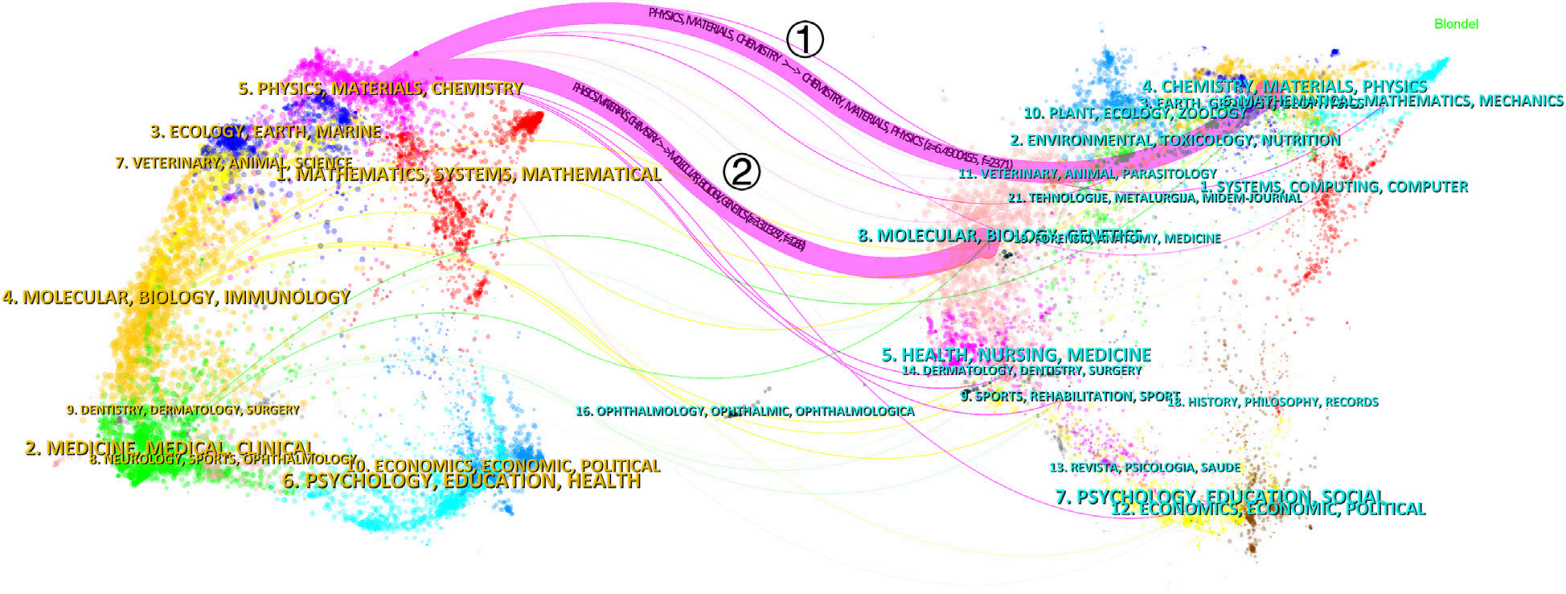
The dual-map overlay of journals related to applications of bioprinted hydrogels in bone regeneration.

The dual-map overlay of journals illustrates the citation relationships between journals and cited journals, with cited journals on the left and cited sources on the right. As shown in Figure 8, two primary and several secondary paths are displayed. Path ① indicates that literature published in CHEMISTRY, MATERIALS, and PHYSICS journals is mainly cited by other literature in PHYSICS, MATERIALS, and CHEMISTRY journals. Path ② indicates that literature published in MOLECULAR, BIOLOGY, and GENETICS journals is primarily cited by papers in PHYSICS, MATERIALS, and CHEMISTRY journals. The top 10 most cited articles in this field are shown in Supplementary Table S3, in which “Three-dimensional (3D) printed scaffold and material selection for bone repair” published by Zhang et al. (2019b) in Acta Biomaterialia in 2019, ranked first with 52 citations.

### 3.6 Factorial analysis and keywords bursts

#### 3.6.1 Factorial analysis

**FIGURE 9:**
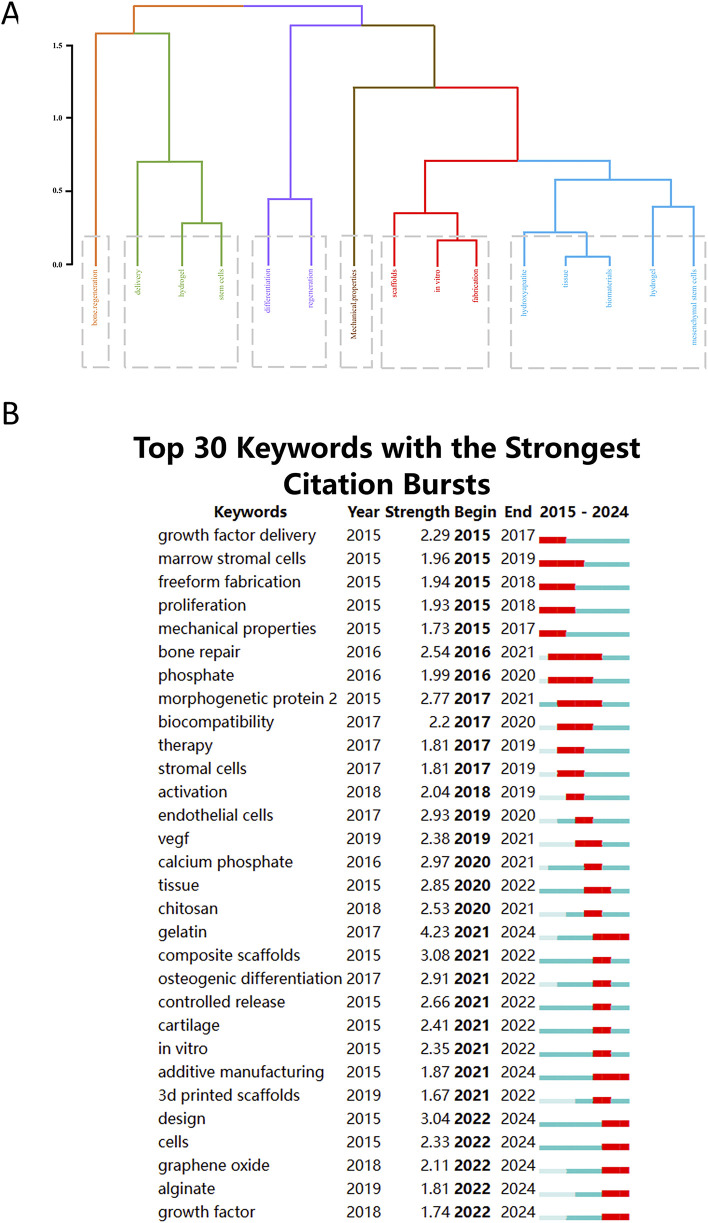
**(A)** The dendrogram shows the most extensive evolution of bioprinted hydrogels in the discipline of bone regeneration. **(B)** Top 30 Keywords with the Strongest Citation Bursts.

We used factorial analysis to identify 6 distinct clusters. As shown in Figure 9A, the categories of bone. regeneration, delivery, hydrogel, stem cells, differentitation, regeneration, Mechanical. properties, scaffolds, *in vitro*, fabrication, hydroxyapatite, tissue, biomaterials, hydrogel, Mesenchymal Stem Cells were not mutually exclusive, with overlap occurring between categories.

#### 3.6.2 Citation burst analysis

We used CiteSpace to generate the top 30 keywords. As shown in Figure 9B, the start and end times of sudden keyword bursts can be visualized, with the shortest yearly interval being 1.67 years. Over the past 15 years, the keywords that have received the most attention for the longest period are “bone repair” (22,016–2021), “phosphate” (2016–2020) and “morphogenetic protein” (2017–2020), all of which have been hotspots for hydrogel applications in bone regeneration. In contrast, keywords that received less attention were “activation” (2018–2019), “endothelial cells” (2019–2020), “composite scaffolds” (2021–2022), “osteogenic differentiation” (2021–2022), “controlled release” (2021–2022) and “*in vitro*” (2021–2022). “gelatin” (2021–2024), “additive manufacturing” (2021–2024), “design” (2022–2024), “cells” (2022–2024), “graphene oxide” (2022–2024), “alginate” (2022–2024) and “growth factor” (2022–2024) are keywords that have been used with high frequency in recent years, suggesting that these aspects may be the focus of research on the application of hydrogels in the bone regeneration field, which has high potential.

## 4 Discussion

Bibliometric analysis is a discipline that responds to the status and trends of research (Chen et al., 2014; Chen et al., 2012). This study provides the first comprehensive analysis of the use of bioprinted hydrogels for bone regeneration and offers statistics on the major journals and organizations in this field.

### 4.1 Research status

Bone is a tissue that can be sustainably regenerated (Dimitriou et al., 2011) and is the hardest organ in the human body. The treatment of both direct and indirect fractures involves bone regeneration. Bone defects not only cause an imbalance in the body, but more importantly, disturb the adaptive system within the bone, limiting its ability to regenerate. The three important processes in bone regeneration are osteoinduction, osteoconduction, and osteointegration (Albrektsson and Johansson, 2001). Osteoinduction refers to the formation of undifferentiated cells for osteogenesis through the aggregation of mesenchymal stem cells (Lewallen et al., 2015), osteoconduction refers to providing an environment for osteoblast growth (Weber, 2019), and osteointegration is the seamless integration of bone tissue with an implant (Morandini Rodrigues et al., 2022). After a patient undergoes trauma and inflammation sets in, mesenchymal stem cells (MSCs) aggregate at the trauma site to form fragile tissue, followed by angiogenesis, which promotes the hardening of soft tissue into hard bone tissue, and finally, osteoclasts and osteoblasts undergo superimposed replacement. Osteoblasts and osteoclasts are key factors involved in the bone regeneration process (Salhotra et al., 2020a). Osteoblasts are mainly derived from stem cells, and MSCs have become common stem cells in the field of bone tissue engineering owing to their proliferative properties (Tsiapalis and O'Driscoll, 2020). Since the internal pore size of the bone varies and is stepped from the inside to the outside, only a suitable pore size can ensure optimal cell adhesion. It has been the most clinically used treatment modality for autologous bone grafting (Dimitriou et al., 2011) because it lacks the risk of immune rejection and retains the same plasticity as living bone (Pereira et al., 2020); however, the limited amount of bone and donor site complications cannot be ignored. Allogeneic grafts have a large numerical advantage but their integration is less efficient (Ehrler and Vaccaro, 2000). Theoretically, allogeneic grafts can achieve the same effects as isografts; however, their use in treatment has not yet been standardized. Artificial bone grafts have become the focus of researchers and are mainly categorized as metals (Lu et al., 2023), bioceramics (Zhao et al., 2022) and polymers (Bharadwaz and Jayasuriya, 2020). However, bone tissue reconstruction with biologically inert metals often leads to secondary surgical repair because of the wear and tear susceptibility of the material (Salhotra et al., 2020b). Compared to other pure bioscaffold materials, hydrogel materials possess unique application potential in the field of bone regeneration.

Hydrogel is a hydrophilic polymer with a three-dimensional network structure, mainly classified as natural (e.g., collagen, hyaluronic acid, chitosan, and alginate) and synthetic (Zhu and Marchant, 2011). The excellent biocompatibility, high mechanical properties, adjustability and cell-carrying properties of hydrogels enable them to better adapt to the geometry and microenvironment of the bone defect sites, while the biodegradable nature of hydrogels avoids the risk of secondary surgeries for patients. Although the excellent biocompatibility of natural hydrogels places them in a high position in the field of regenerative medicine, their potential uncontrollability is an issue that researchers cannot ignore. Synthetic hydrogels lack biological activity, although they exhibit strong mechanical properties. Consequently, researchers are constantly searching for hydrogel materials that can carry cells. Saravanan et al. (Saravanan et al., 2018) indicated that CS/GP/GO hydrogels have better biocompatibility with MSCs and can promote osteogenesis, while Liu et al. (Liu et al., 2020) indicated that CD/HA/PVA hydrogels can support the proliferation and differentiation of MSCs *in vivo*.

Recently, bioprinting technology, which enables the construction of 3D structures using cells, proteins, and biomaterials, has been applied in the field of bone regeneration (Dimitriou et al., 2011) and offers a possibility for bone regeneration therapy. Multi-layer stacked printing can simulate the internal complexity of natural bone tissue, individualize the geometry of the area to be filled with bone defects, and adjust the hydrogel properties to match the mechanical strength of natural bone. Breakthroughs have been made in 3D bioprinting technology (Daly et al., 2017), and cell-loaded bioprinting has been realized (Yang et al., 2017). Bioprinted structures can change the internal spatial structure at different stimuli, enabling a high degree of control over mesenchymal stem cells. However, owing to the limitations of 3D bioprinting in personalizing the treatment of bone defects, 4D bioprinting was developed. This technology can change over time in response to stimuli such as pH, temperature, and is well adapted to the microenvironmental reconstruction of irregular bone defect sites (Zhang et al., 2019a). It can fulfill the functional transformation between organisms and hydrogel materials. In addition, bioprinted hydrogel scaffolds enable controlled release of bioactive molecules to induce vascularization and nerve regeneration in regenerated bone. Currently, bioprinted hydrogel scaffolds applied in the field of bone regeneration have been able to achieve good physical support from the macroscopic structure, but how to change the bionic properties of the microstructure of the hydrogel material to match different functional characteristics is the current challenge. So bioprinting of in situ-loaded stem cell bone-like organs may be the future development direction.

The results of the Most Relevant Affiliations indicate that research on the application of bioprinted hydrogels in bone regeneration is still in its initial stage, proving the great potential of research in this field. The results of the analysis of cooperation between institutions and countries show that although the cooperation between China and the United States is closer, which provides favorable conditions for technological exchanges between the two countries. However, the cooperation network between China and other countries is relatively weaker. The cross-regional and cross-institutional cooperation needs to be strengthened, therefore, institutions of various countries should quickly establish excellent academic cooperation in order to promote the rapid development of bioprinting hydrogel technology in the field of bone regeneration.

### 4.2 Research hotspots and prospects

Biomaterials science combined with stem cell therapy and tissue engineering techniques are the basis of regenerative medicine. Bone regenerative medicine is a research area that focuses on bioprinted hydrogels. Compared with traditional techniques, bioprinted hydrogels can maximize the filling of space at the fracture site while simultaneously guaranteeing the stability of their mechanical properties. In recent years, research has shown on the development of cell-loaded hydrogels for the reconstruction of bone tissue for vascularization and nerve regeneration (Ashammakhi et al., 2019). However, the desirable decellularized extracellular matrix (ECM) of bone tissue has special mechanical structure and biochemical signals that can support cell adhesion and proliferation, Therefore, achieving the structural stability and signal maintenance of the ECM during decellularization in bioprinted hydrogels is the direction of scholars’ further research. Additionally, exosome hydrogels should also be capable of promoting new vessel generation and tissue regeneration *in vivo*, as well as inhibiting local tissue fibrosis.

In addition, 3D bioprinting enables the control of hydrogel structure and function, while 4D bioprinting further alters the morphology of the hydrogel over time. Future research on bioprinting will focus on the spatial equilibrium of MSCs with hydrogel scaffolds and osteogenic transformations (Benning et al., 2017). Bioprinting is a relatively recent technology (Kumar Gupta et al., 2022). Although bioprinting technology has been widely used in the field of biomedical engineering, bioprinted hydrogels have certain limitations in terms of computer program settings, large-scale production, and cell-carrying efficiency. To prevent the reduction of cell viability in printed hydrogels, the release of alkaline ions (Heid and Boccaccini, 2020) to prevent local pH increases or to optimize the thickness of the hydrogel coating (Ringeisen et al., 2004) can be considered. Bioprinting can improve the precision of personalized treatment for bone defects to a certain extent. However, printing standardization needs further exploration. To ensure the sustainable development of this technology in the medical field, future restrictions need to be strengthened in terms of the relevant requirements of regulatory authorities, demonstration of safety and efficacy, and translation of results. The stability and safety of hydrogels for bioprinting have to be demonstrated in a variety of animal models to further characterize their physicochemical properties such as degradability and mechanical properties. Additionally, qualified materials and standardized manufacturing lines during clinical translation are prerequisites for avoiding immune response. There is an immediate demand for further biological optimization of the materials and standard safe operating procedures. Basic research is the cornerstone of the development of bone regeneration technology, and translating the results is the key to clinical treatment. Reducing the cost of bioprinting, improving hydrogel cell-carrying technology, and shortening the time required for bone regeneration will promote the application of alternative hydrogel bone grafts in clinical treatment in the field of bone regeneration in the future.

## 5 Conclusion

Over the past 15 years, the use of bioprinted hydrogels in the field of bone regeneration has continued to attract attention. In this study, 684 documents collected from the Web of Science database were processed and visualized for data analysis using bibliometric tools. The numerous articles will be analyzed objectively, systematically, and comprehensively to enhance the reader’s perspective on the field as a whole. The study shows that the main institutions involved in the field of bone regeneration globally are concentrated in China and the United States. China ranks first in the field of bone regeneration in terms of the number of publications and the number of SCPs, with close cooperation between China and the United States. The journal of *Biomaterials* is the most published journal in this field, and the article “3D bioactive composite scaffolds for bone tissue engineering” by TURNBULL G et al. in *Bioactive Materials* is the most cited article in the field. These studies provide a rapid and precise orientation for researchers who are about to carry out studies in this field. At the same time, they provide a reference for future interdisciplinary collaborations. In addition, these findings can help policymakers in the industry to take a comprehensive and systematic view of the field’s growth prospects. However, this study, which is based on previous research with a certain lag, has some limitations. We only collected published papers, not those that will be published or are still undergoing research, ignoring some potentially valuable papers.

## Abbreviations

SCP, Single-country Publications; MCP, Multi-country Publications; CaP, Calcium Phosphate; 3D, Three-dimensional; 4D, Four-dimensional; SCU, Sichuan University; SJTU, Shanghai Jiao Tong University; MSCs, Mesenchymal Stem Cells; ECM, Extracellular Matrix.
