# Supplementary material for: Bioprinted hydrogels in bone regeneration: a bibliometric analysis
Source: Front Pharmacol. 2025 Feb 3;16:1532629. doi: 10.3389/fphar.2025.1532629 (PMC11830744; doi:10.3389/fphar.2025.1532629)
Supplement: Supplementary file 1 [file Table1.docx]

Supplementary Table S1. Top 10 authors with the highest number of posts.

| Rank | Authors | Count | year | % of 684 |
| --- | --- | --- | --- | --- |
| 1 | Zhou, Xiaojun | 12 | 2020 | 1.7 |
| 2 | He, Chuanglong | 10 | 2020 | 1.4 |
| 3 | Fan, Yujiang | 9 | 2020 | 1.3 |
| 4 | Lin, Tao | 9 | 2019 | 1.3 |
| 5 | Liu, Chun | 9 | 2022 | 1.3 |
| 6 | Shao, Huiping | 9 | 2019 | 1.3 |
| 7 | Zhang, Xingdong | 9 | 2020 | 1.3 |
| 8 | Peng, Jiang | 8 | 2019 | 1.1 |
| 9 | Liu, He | 7 | 2019 | 1.0 |
| 10 | Liu, Xiao | 7 | 2023 | 1.0 |

Supplementary Table S2. Top 10 keywords with the most words appearing in Bioprinted Hydrogel in Bone Regeneration.

| Rank | Keywords | Centrality | Count |
| --- | --- | --- | --- |
| 1 | bone regeneration | 0.01 | 226 |
| 2 | 3d printing | 0.1 | 189 |
| 3 | scaffolds | 0.02 | 132 |
| 4 | hydrogels | 0.02 | 118 |
| 5 | mesenchymal stem cells | 0.11 | 114 |
| 6 | hydrogel | 0.08 | 104 |
| 7 | tissue engineering | 0.06 | 102 |
| 8 | in vitro | 0.05 | 101 |
| 9 | differentiation | 0.07 | 86 |
| 10 | bone tissue engineering | 0.14 | 84 |

Supplementary Table S3. Top 10 Most Cited Articles on Bioprinted Hydrogels in Bone Regeneration.

| Rank | Cited references | DOI | Year | Count |
| --- | --- | --- | --- | --- |
| 1 | Zhang L, 2019, ACTA BIOMATER, V84, P16 . | DOI 10.1016/j.actbio.2018.11.039 | 2019 | 52 |
| 2 | Koons GL, 2020, NAT REV MATER, V5, P584. | DOI 10.1038/s41578-020-0204-2 | 2020 | 37 |
| 3 | Kang HW, 2016, NAT BIOTECHNOL, V34, P312. | DOI 10.1038/nbt.3413 | 2016 | 32 |
| 4 | Zhang M, 2020, SCI ADV, V6, P0. | DOI 10.1126/sciadv.aaz6725 | 2020 | 31 |
| 5 | Wang C, 2020, BIOACT MATER, V5, P82. | DOI 10.1016/j.bioactmat.2020.01.004 | 2020 | 31 |
| 6 | Yan YF, 2019, BIOMATERIALS, V190, P97. | DOI 10.1016/j.biomaterials.2018.10.033 | 2019 | 29 |
| 7 | Turnbull G, 2018, BIOACT MATER, V3, P278. | DOI 10.1016/j.bioactmat.2017.10.001 | 2018 | 29 |
| 8 | Matai I, 2020, BIOMATERIALS, V226, P0. | DOI 10.1016/j.biomaterials.2019.119536 | 2020 | 23 |
| 9 | Murphy SV, 2014, NAT BIOTECHNOL, V32, P773. | DOI 10.1038/nbt.2958 | 2014 | 23 |
| 10 | Ashammakhi N, 2019, ADV HEALTHC MATER, V8, P0. | DOI 10.1002/adhm.201801048 | 2019 | 23 |
